# Data of self-made Taq DNA polymerase prepared for screening purposes

**DOI:** 10.1016/j.dib.2017.03.004

**Published:** 2017-03-09

**Authors:** E.V. Konovalova, A.A. Schulga, T.I. Lukyanova, E.J. Woo, S.M. Deyev

**Affiliations:** a Shemyakin-Ovchinnikov Institute of Bioorganic Chemistry RAS, Moscow, Russia; b Disease Target Structure Research Center, Korea Research Institute of Bioscience and Biotechnology, Daejeon 305-333, South Korea

**Keywords:** Taq, Polymerase chain reaction, Screening, Analysis, Colonies, Genomic, Chromosomal DNA

## Abstract

DNA analysis is a key procedure in genetic engineering. Nowadays the analysis is often done by PCR with Taq DNA polymerase. Although the last enzyme price is quite low, demand for numerous analyses results in much money expenditure which are not affordable for many laboratories. In a meanwhile, many screening tasks do not require the highly purified enzyme. Taking into account the enzyme unique properties it makes possible to marginally simplify its production without resorting to costly or lengthy techniques such as column chromatography and/or dialysis. Here the data of routine usage of Taq DNA polymerase prepared according to the protocol developed in our laboratory is presented. The protocol takes only several hours to realize and does not need qualified personnel or expensive equipment. Yet it gives the enzyme preparation suitable for most screening purposes. The isolated Taq DNA polymerase stock can be stored as ammonium sulfate suspension in a refrigerator for prolonged period, not less than 6 months. The working enzyme solution is prepared from the stock suspension on demand, not more than once in a month and can be stored also in a refrigerator.

**Specifications Table**

**Table:** 

Subject area	*Molecular biology*
More specific subject area	*Genetic engineering*
Type of data	*Text, figures*
How data was acquired	*SDS-polyacrylamide gel electrophoresis, agarose gel electrophoresis, Bio-Rad T100 thermal cycler*
Data format	*Raw, analyzed*
Experimental factors	*Taq polymerase stability, PCR analysis*
Experimental features	*Taq polymerase isolation, bacterial colonies screening*
Data source location	*Moscow, Russia*
Data accessibility	*The data is in this article*

**Value of the data**

– Simple protocol of self-made Taq DNA polymerase production and usage.
– Convenient way of the self-made enzyme storage in a refrigerator without freezing.
– The data of the self-made enzyme stability during long-term storage in a refrigerator.
– The data of PCR analyses with the self-made enzyme for many samples.
– Significant reduction of PCR analyses expenses.

## 1 Data

**Fig. 1:**
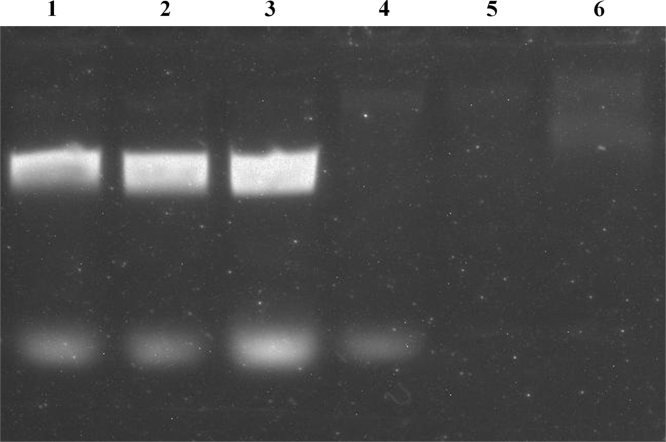
Analysis of Taq DNA polymerase activity in the enzyme stock fractions: (1–3) the PCR reactions with 0.5 µl, 1 µl and 2 µl of the working enzyme solution, (4–6) the PCR reaction with 0.5 µl, 1 µl and 2 µl of the AS suspension supernatant.

**Fig. 2:**
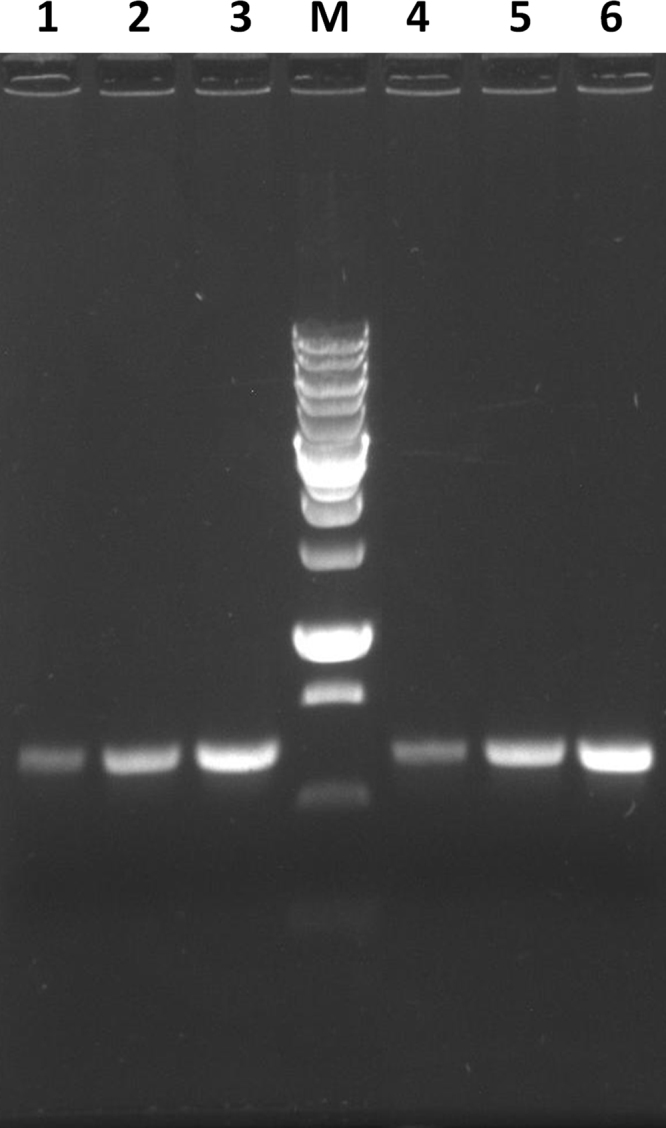
The working enzyme solution stability test: (1–3) the PCR reactions on bacterial chromosome with 0.5 µl, 1 µl and 2 µl of the working enzyme solution stored a month in a refrigerator; (4–6) the PCR reactions with 0.5 µl, 1 µl and 2 µl of the freshly prepared working enzyme solution; lane M contains the DNA ladder (Sibenzyme, #M11): 0.25, 0.5, 0.75, 1 (more intense fragment), 1.5, 2, 2.5, 3 (more intense fragment), 4, 5, 6, 8, 10 kbp.

The Taq DNA polymerase was isolated as described in [1] with some modifications making the purification protocol simpler and more convenient. First, the recombinant strain biomass was obtained by inoculation of auto-induction medium with bacterial colonies from agar plate [2]. Second, the isolated Taq DNA polymerase stock was stored as ammonium sulfate (AS) suspension in a refrigerator (at 4–6 °C) – not less than 6 months. The working enzyme solution was prepared in small portions, as required, by AS suspension centrifugation and the precipitate solubilization in a standard 1×Taq buffer. The working enzyme solution was stored in a refrigerator (at 4–6 °C) not less than a month. On Fig. 1 there is data of the Taq DNA polymerase activity in the AS suspension fractions. The data of the working enzyme solution stability is presented on Fig. 2. For the analyses a common master mix containing 1×Taq buffer, primers, dNTPs, and a small amount of BL21(DE3) biomass was prepared. The primers were annealed on the BL21(DE3) chromosome 625 bp apart.

**Fig. 3:**
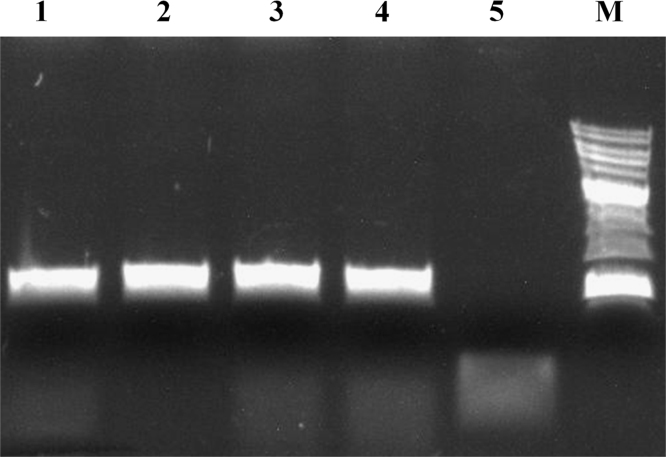
A typical analysis of five *E. coli* plasmid-containing colonies. (1–5) PCR reactions; (6) DNA ladder (Sibenzyme, #M11): 0.25, 0.5, 0.75, 1 (more intense fragment), 1.5, 2, 2.5, 3 (more intense fragment), 4, 5, 6, 8, 10 kbp. Colonies giving the PCR fragment of about 1 kb in length contained the desired insert.

On Fig. 3 there is typical PCR analysis of five plasmid-containing bacterial colonies. The scheme of the experiment was as follows. The DNA fragment of about 900 bp was amplified by PCR and cloned in commercial pAL2-T vector (Evrogen). The universal oligonucleotide primers M13dir/M13rev and the working enzyme solution were used for PCR analysis. Four of 5 colonies were shown to contain the desired insert. The data was verified by sequencing from M13dir and M13rev primers (Evrogen).

**Fig. 4:**
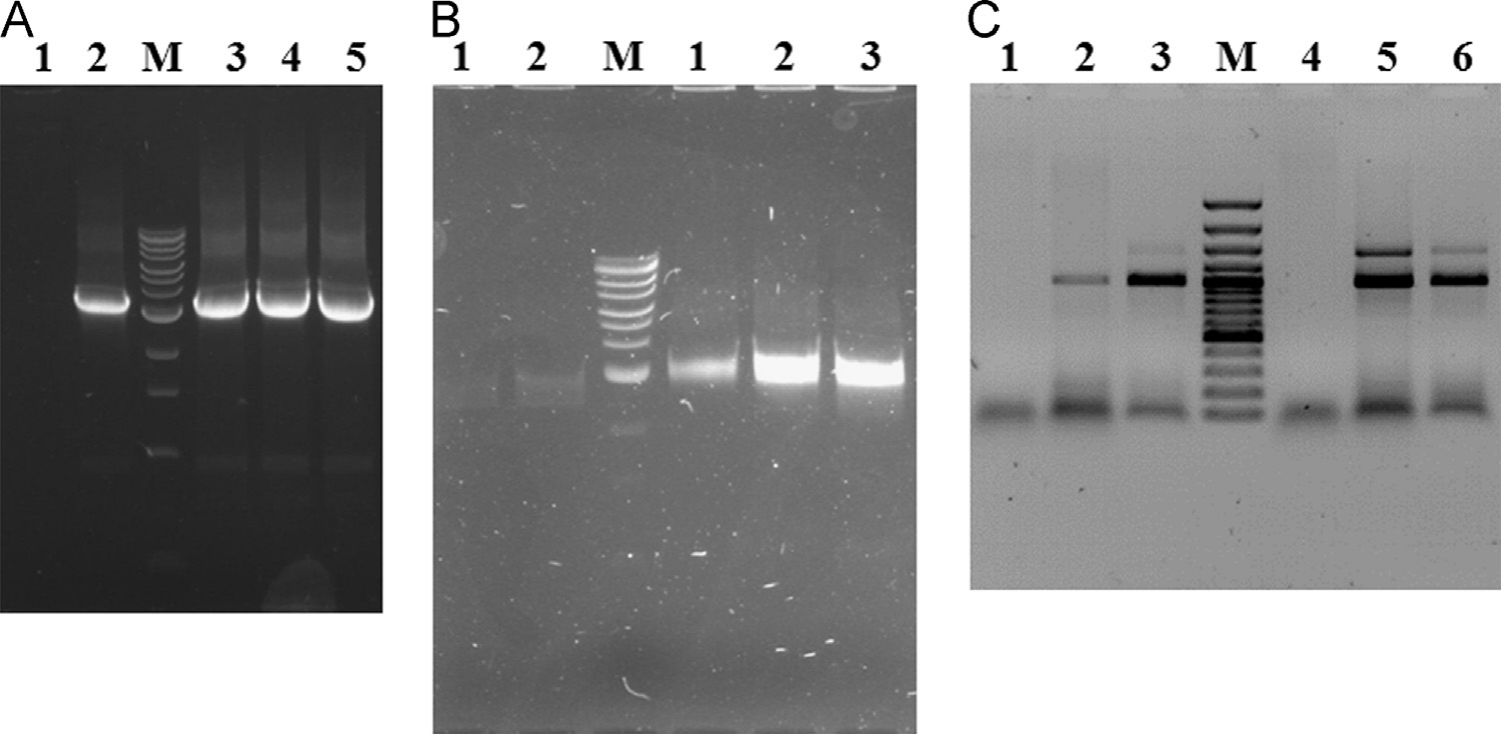
Comparative PCR analyses of different samples with the self-made enzyme and the commercial ones. PCR analyses of the same DNA fragment as a part of a plasmid (A) and bacterial chromosome (B). The reactions contained (1) 2.5 u and (2) 5 u of Taq DNA polymerase supplied by Evrogen; (3) 0.5 μl, (4) 1 μl and (5) 2 μl of the self-made enzyme. Lane M contains the DNA ladder (New England Biolabs, #N3239S): 0.5, 1, 1.5, 2, 3 (more intense fragment), 4, 5, 6, 8, 10, 15, 20, 48.5 kbp. A common master mix was prepared for all samples. (C) PCR analyses of two cassettes incorporated in a binary plasmid DNA (3, 6) and in genomic DNA of transgenic chrysanthemum plant (2, 5). Lane M contains the DNA ladder (Thermo Fisher Scientific, #SM0321): 100, 200, 300, 400, 500 (more intense fragment), 600, 700, 800, 900, 1000 (more intense fragment), 1200, 1500, 2000, 3000 bp. On the left from lane M there are the reactions which contained 1 u of Thermo Fisher Scientific Taq DNA polymerase (#EP0402). On the right from the lane M there are the reactions which contained 1 μl of the self-made enzyme. Lanes (1 and 4) contains the control reactions without addition of DNA. A common master mix was prepared for all samples.

On Fig. 4 there is data of comparative analysis of different samples with the self-made Taq DNA polymerase and the commercial ones supplied with Evrogen (Russia) and Thermo Fisher Scientific (USA). The same DNA fragment containing the expression cassette for the bacillar RNase barnase along with the kanamycin resistance gene was analyzed with flanking primers as part of plasmid (Fig. 4A) and bacterial chromosome (Fig. 4B). The size of the PCR product was about 3.2 kb that corresponded well with the expected size. The data of PCR analysis of two cassettes: 35S: hptII: pA35 and 35S: HAM75: pA35 incorporated in a binary plasmid DNA and in genomic DNA of transgenic chrysanthemum plant with 35S- and pA35-specific primers is presented on Fig. 4C. The expected sizes of the PCR products are about 960 and 1450 bp. Both PCR products are revealed in the reaction.

## 2 Experimental design, materials and methods

### 2.1 Taq DNA polymerase stock preparation

Bacteria BL21(DE3) were transformed with plasmid pTaq containing the Taq DNA polymerase gene under the transcriptional control of lac promoter and spread over an agar plate with ampicilline (100 µg/ml) which was incubated at 37 °C overnight. Auto-induction medium ZYM-5052 [2] (50 ml) was inoculated with several colonies from the plate. The culture was grown in the thermostatic shaker at 37 °C and 200 rpm overnight. The bacteria cells were harvested by centrifugation at 7000 *g* for 15 min. The *Taq* isolation method was based essentially on [1]. The biomass was resuspended in 10 ml of 100 mM Tris–Cl, 0.1 mM PMSF, pH 8.0. The egg lysozyme was added at 60 µg/ml. After mixing the equal volume of water was added. The suspension was incubated at room temperature for 30 min, and then treated with ultrasound for 10 min (treatment 10 sec, pause 10 sec). Tween-20 was added to final concentration 0.25%, Nonidet P40 – to 0.25% and KCl – to 25 mM. The suspension was distributed into 2 ml tubes and heat treated at 75 °C for 1 h with occasional overturning, then spun at 20 000 *g* and 4 °C for 30 min in a refrigerated Eppendorf centrifuge. The supernatant was collected in a small glass beaker. The ammonium sulfate (AS) powder was added to final concentration 30%. The resultant suspension is called as the Taq DNA polymerase stock. The stock can be stored in a refrigerator at 6–8 °C for several months, at least. For example, the data presented on Fig. 4 was obtained with the enzyme stock of 7 months old. The quantity of Taq DNA polymerase prepared in such a way is sufficient for 5000 PCR reactions.

### 2.2 The working enzyme solution preparation

The enzyme stock was thoroughly stirred. The suspension in a volume of 200 μl was transferred to 1.5 ml tube and spanned at 12 000 *g* for 5 min. The supernatant was carefully removed. The precipitate was dissolved in 50 µl of 1×Taq standard buffer. The tube was centrifuged at 12 000 *g* for 5 min. The supernatant was transferred into a fresh tube and stored in a refrigerator at 6–8 °C until finished (not less than 1 month). The working solution should be prepared regularly, as required, because it is more labile than AS suspension.

The working enzyme solution specific activity was determined in comparison with the commercial Taq DNA polymerase preparations (Fig. 4). The reactions were carried out in identical conditions. Accordingly, a common master mix for the two enzymes was prepared, which was distributed into reaction tubes. Then different volumes of the tested enzymes were added into each tube. Usually one microliter of the working enzyme solution was sufficient for any PCR analysis. It corresponded to about 5 activity units of the Evrogen׳s enzyme (#PK013S) or to about 1 activity unit of the Thermo Fisher Scientific enzyme (#EP0402) (Fig. 4).

### 2.3 PCR analyses

A common master mix was prepared to analyze all samples. Final concentrations of components in the reaction were 10 mM Tris–HCl, 50 mM KCl, 1.5 mM MgCl_2_, pH 8.3, 4 pmoles each of primers, 0.2 mM dNTPs. For each analysis 1 µl of the working enzyme solution was added.

#### 2.3.1 Bacterial colonies analysis

The bacterial colonies were picked up by a sterile 200 µl tip and added into the PCR reaction.

#### 2.3.2 Bacterial biomass analysis

The biomass equivalent to 1 µl of liquid culture was added to each reaction.

#### 2.3.3 Plasmid DNA analysis

1–2 ng of plasmid DNA was added per reaction.

#### 2.3.4 Plant chromosome analysis

150 ng of plant genomic DNA was added per reaction.

PCR reactions were carried out in a final volume of 20 µl with Bio-Rad T100 thermal cycler. The program was set as follows: the lid temperature is 104 °С, 94 °C for 1.5 min for the initial denaturation, a 3-step temperature cycling 20 times between 94 °C for 15 s, X °C for 15 s and 68 °C for *t* min depending on the primers and target size. The annealing temperature was 5 °C below the lowest primer′s melting temperature [3]. The extension time was one minute per 1000 base pairs. In the case of *E. coli* chromosomal DNA analysis the number of cycles was increased to 30 and in the case of plant genomic DNA analysis – to 40.
